# Ethnic bias and clinical decision-making among New Zealand medical students: an observational study

**DOI:** 10.1186/s12909-018-1120-7

**Published:** 2018-01-23

**Authors:** Ricci Harris, Donna Cormack, James Stanley, Elana Curtis, Rhys Jones, Cameron Lacey

**Affiliations:** 1 Te Kupenga Hauora Māori, Faculty of Medical and Health Sciences, University of Auckland, Private Bag 92019, Auckland, 1142 New Zealand; 2 Biostatistics Group, Dean’s Department, University of Otago Wellington, PO Box 7343, Wellington, 6242 New Zealand; 3 Māori/Indigenous Health Institute (MIHI), University of Otago Christchurch, PO Box 4345, Christchurch, 8140 New Zealand

**Keywords:** New Zealand, Racial/ethnic bias, Medical students, Māori, Clinical decision-making, Implicit association test, Vignettes, Bias and Decision-Making in Medicine (BDMM) study

## Abstract

**Background:**

Health professional racial/ethnic bias may impact on clinical decision-making and contribute to subsequent ethnic health inequities. However, limited research has been undertaken among medical students. This paper presents findings from the *Bias and Decision-Making in Medicine (BDMM)* study, which sought to examine ethnic bias (Māori (indigenous peoples) compared with New Zealand European) among medical students and associations with clinical decision-making.

**Methods:**

All final year New Zealand (NZ) medical students in 2014 and 2015 (*n* = 888) were invited to participate in a cross-sectional online study. Key components included: two chronic disease vignettes (cardiovascular disease (CVD) and depression) with randomized patient ethnicity (Māori or NZ European) and questions on patient management; implicit bias measures (an ethnicity preference Implicit Association Test (IAT) and an ethnicity and compliant patient IAT); and, explicit ethnic bias questions. Associations between ethnic bias and clinical decision-making responses to vignettes were tested using linear regression.

**Results:**

Three hundred and two students participated (34% response rate). Implicit and explicit ethnic bias favoring NZ Europeans was apparent among medical students. In the CVD vignette, no significant differences in clinical decision-making by patient ethnicity were observed. There were also no differential associations by patient ethnicity between any measures of ethnic bias (implicit or explicit) and patient management responses in the CVD vignette. In the depression vignette, some differences in the ranking of recommended treatment options were observed by patient ethnicity and explicit preference for NZ Europeans was associated with increased reporting that NZ European patients would benefit from treatment but not Māori (slope difference 0.34, 95% CI 0.08, 0.60; *p* = 0.011), although this was the only significant finding in these analyses.

**Conclusions:**

NZ medical students demonstrated ethnic bias, although overall this was not associated with clinical decision-making. This study both adds to the small body of literature internationally on racial/ethnic bias among medical students and provides relevant and important information for medical education on indigenous health and ethnic health inequities in New Zealand.

**Electronic supplementary material:**

The online version of this article (10.1186/s12909-018-1120-7) contains supplementary material, which is available to authorized users.

## Background

The potential contribution of health providers’ racial/ethnic bias to ethnic health inequities is an area receiving increasing research attention [1–3]. Bias can be defined as “... generally negative feelings and evaluations of individuals because of their group membership (prejudice), overgeneralized beliefs about the characteristics of group members (stereotypes), and inequitable treatment (discrimination)” [3], p201. Within this, explicit bias is considered “conscious and intentional” [3], p201, and implicit bias “unconscious and automatically activated” [3], p201. Racial/ethnic bias exists within a broader system of racism that structures opportunity, resources and power by race/ethnicity, with multifarious expressions that impact on health in many ways [4], and is one possible manifestation of this broader system [2].

Studies examining health professional racial/ethnic bias to date are largely US based and focused on pro-White/anti-Black race bias [5–7]. They consistently demonstrate pro-white racial/ethnic bias among health professionals, although relationships to healthcare vary [5–7]. Implicit racial bias has been shown to impact on the quality of healthcare encounters [8], and on clinical decision-making [9, 10], although not consistently [11–15]. Within this field, most studies have been undertaken among physicians, with very few among medical students [12, 16, 17]. In a study of 211 students entering a US medical school, the majority of participants (54%) demonstrated an implicit preference for ‘white’ (vs ‘black’) people [12]. Explicit preference for ‘white’ people was also present (39% of participants) although not as pronounced as implicit preference. No consistent differences were found in vignette assessment by patient race or in the relationship between racial/ethnic bias and clinical assessment in this study [12]. In another US study of students entering medicine, nursing and pharmacy, medical students (along with nursing and pharmacy students) demonstrated an implicit preference for ‘Whites’ (compared to ‘Blacks’) and a preference for lighter (compared to darker) skin tone [16]. In a study of third year medical students, Gonzales et al. [17] also demonstrated that medical students were more likely to have an implicit “preference for people like themselves” (p66) in a sample where the majority of students were ‘White’.

In 2013, the New Zealand (NZ) population was 4.4 million with 14% Māori (the indigenous population) and 70% European [18]. Major inequities exist for most health measures, including healthcare access and quality, between Māori and non-Māori [19, 20]. Māori report higher experience of racial discrimination by health professionals compared to Europeans [21], with qualitative evidence of negative beliefs and stereotypes about Māori among health professionals [22, 23]. The current project explores ethnic bias and clinical decision-making amongst medical students, as part of a broader project on how medical education can address indigenous health inequities. As with all health professionals, medical students in NZ are likely to be exposed to a range of discourses and stereotypes about Māori, both within medical education and in broader society. Research is needed to expand the body of evidence of racial/ethnic bias among medical students in general. Understanding medical student bias may provide for improved learning environments for both students and teachers and reduce future impacts of racial/ethnic bias on ethnic health inequities. It is important that this occurs within and across country contexts, in order to capture the similarities and differences in how racial/ethnic bias may operate for different populations and in different health systems. This research will also provide relevant information to support medical education in particular locations. In addition, the examination of bias and the implications for Māori health and ethnic inequities in New Zealand provides information from an indigenous health perspective that is internationally relevant and for which studies are also limited [14, 24].

This study sought to (1) measure ethnic bias towards Māori compared with NZ European among final year medical students, (2) assess differences in clinical decision-making responses to chronic disease vignettes for Māori versus NZ European patients, and (3) examine associations of implicit and explicit ethnic bias with clinical decision-making. Given the evidence of ethnic healthcare inequities between Māori and non-Māori, and negative beliefs about Māori held by healthcare professionals (outlined above), we hypothesized (a priori) that medical students would exhibit ethnic bias favoring NZ European, differentially respond to management decisions by patient ethnicity, and that ethnic bias would be associated with differential management by patient ethnicity.

## Methods

### Study design

A web-based cross-sectional study was undertaken with final year NZ medical students in two consecutive academic years. Study development, pretesting, piloting and final questionnaire are detailed elsewhere [25]. The study was approved by the University of Auckland Human Participants Ethics Committee (Reference 011693) and ratified by the University of Otago Human Ethics Committee.

### Participants and data collection

Email invitations and a password to enter the study were sent to all final year medical students via student administrators at the Universities of Otago and Auckland (*n* = 888) in November 2014 (*n* = 438) and January 2015 (*n* = 450). For each group, the study website was open for 12 days during which two to three reminder emails were sent.

On entering the study website, after reading an information sheet, participants were required to consent in order to proceed. Participation was anonymous and voluntary. Participants were offered a $20 electronic gift voucher and the chance to enter a draw for a $500 gift voucher.

In order to minimize bias from knowledge of the study’s purpose, initial information did not specify a focus on ethnic bias, although additional information was provided at the end of the questionnaire. To minimize social desirability bias [26] study content was ordered as: key demographics; vignettes; two implicit bias measures; social desirability scale; explicit bias measures; and, additional demographic questions. As explicit bias may be more prone to participants providing socially acceptable responses [7], we placed this module last so as not to influence the responses to the other modules.

302 students (34% of invited students) entered the study. 287 participants answered at least one vignette module, while 280 participated in both. Participant characteristics were proportionally similar to the invited student group by age, gender and ethnicity (Table 1).

**Table 1:** Participant characteristics by vignette patient ethnicity

		CVD vignette patient ethnicity		Depression vignette patient ethnicity			
		NZ Euro	Māori	NZ Euro	Māori	Total	Total sample characteristics
Participant Characteristics	Level	n (%)	n (%)	n (%)	n (%)	n (%)	(%)
		Total *n* = 145	Total *n* = 142	Total *n* = 142	Total *n* = 138	Total *n* = 287	Total *n* = 888
Prioritized Ethnicity	European	76 (52)	93 (65)	88 (62)	77 (56)	169 (59)	(51)
	Māori	8 (6)	8 (6)	7 (5)	9 (7)	16 (6)	(9)
	Pacific	6 (4)	3 (2)	3 (2)	5 (4)	9 (3)	(5)
	Asian	54 (37)	34 (24)	42 (30)	44 (32)	88 (31)	(32)
	Other	1 (1)	4 (3)	2 (1)	3 (2)	5 (2)	(3)
Age	Median (IQR)	23 (23–24)	24 (23–25)	24 (23–25)	23 (23–25)	24 (23–25)	24
	Missing	2 (1)	1 (1)	2 (1)	1 (1)	3 (1)	
Gender	Male	65 (45)	71 (50)	68 (48)	66 (48)	136 (47)	(47)
	Female	80 (55)	71 (50)	74 (52)	72 (52)	151 (53)	(53)
Self-reported SES	Low	2 (1)	6 (4)	2 (1)	6 (4)	8 (3)	
	Lower-middle	22 (15)	24 (17)	25 (18)	21 (15)	46 (16)	
	Middle	48 (33)	36 (25)	43 (30)	41 (30)	84 (29)	
	Upper-middle	44 (30)	44 (31)	46 (32)	42 (30)	88 (31)	
	High	7 (5)	8 (6)	9 (6)	6 (4)	15 (5)	
	Not reported^b^	22 (15)	24 (17)	17 (12)	22 (16)	46 (16)	
Born in NZ	Yes	69 (48)	82 (58)	78 (55)	73 (53)	151 (53)	
	No	54 (37)	36 (25)	47 (33)	43 (31)	90 (31)	
	Not reported^b^	22 (15)	24 (17)	17 (12)	22 (16)	46 (16)	
SDRS^a^	0	54 (37)	69 (49)	57 (40)	66 (48)	123 (43)	
	1	42 (29)	27 (19)	37 (26)	32 (23)	69 (24)	
	2	18 (12)	15 (11)	21 (15)	12 (9)	33 (11)	
	3	9 (6)	6 (4)	10 (7)	5 (4)	15 (5)	
	4	0 (0)	0 (0)	0 (0)	0 (0)	0 (0)	
	5	1 (1)	1 (1)	0 (0)	2 (1)	2 (1)	
	Not reported^b^	21 (14)	24 (17)	17 (12)	21 (15)	45 (16)	

^a^SDRS Social Desirability Response set (0 low social desirability to 5 high social desirability)

^b^Frequencies for ‘Not reported’ represent individuals who did not reach the final section of the survey tool where these questions were asked, plus one individual who did not complete all questions in the final section

### Key variables

#### Chronic disease vignettes

Two existing clinical vignettes focused on cardiovascular disease (CVD) and possible depression [9, 27] were adapted with permission for the NZ context, using a process of clinical review and revision [25]. Participants were asked about diagnosis and management for the vignette patients [9, 25, 28]. To examine differential responses by patient ethnicity, the vignettes described patient ethnicity as either Māori or NZ European, with common English or corresponding Māori language surnames used as additional ethnicity ‘markers’. The particular patient ethnicity seen for the two vignettes, and the order of these vignettes for a given participant, was randomized so as to balance these factors across all participants (e.g. so that an equal number of Māori and NZ European CVD vignettes were seen across all participants; and that CVD and depression vignettes were equally often seen in the first position, rather than CVD always preceding the depression vignette).

The cardiovascular disease (CVD) vignette described a 50-year-old man presenting to a regional hospital emergency department with chest pain and ECG changes suggestive of myocardial infarction (MI). Participants were asked to rate the likelihood that the pain was due to coronary artery disease (responses on a 5-point scale, 1 = very unlikely to 5 = very likely), and their recommendation in relation to thrombolysis (1 = would definitely recommend thrombolysis to 5 = would definitely NOT recommend thrombolysis) [9]. Thrombolysis responses were reverse scored for analysis. Following additional information and instructions to give thrombolysis for a likely MI, participants were asked about their subsequent management if the patient refused thrombolysis (1 = ‘I would not try to persuade him any further’ to 5 = ‘I would try very hard to persuade him’) [9].

The depression vignette described a 44-year-old man presenting to his general practitioner (family physician) with generalized symptoms including muscle aches, insomnia, headache, irritability and reduced concentration, and a past history (25 years prior) of depression. Participants were asked to rate the likelihood that the patient’s symptoms were due to depression (1 = very unlikely to 5 = very likely) [9], the severity of the patient’s symptoms (1 = none-slight, 2 = mild, 3 = moderate, 4 = severe) and to rank from 1 to 5 their preferred initial management approach from a list of options: a) encourage self-help strategies; b) refer to social support and counseling services; c) recommend anti-depressant treatment; d) refer to primary care brief intervention service; and, e) commence anti-depressant and refer to specialist mental health services) [25]. Participants also rated the likelihood that the patient would benefit from the selected initial management approach (1 = very unlikely to 5 = very likely) [28].

#### Implicit bias measures

The implicit association test (IAT) is well validated [29] and the most commonly used implicit bias measure in studies examining racial/ethnic bias among health professionals [5, 6]. The IAT is a computer based response latency measure where participants have to rapidly sort stimuli into target categories (e.g. ethnicity) and attitude or stereotype categories [29]. The IAT measures the relative strength of association between the contrasted groups with respect to how quickly different pairs of stimuli are sorted [30]. Two IATs were included, an ‘ethnicity preference IAT’ and an ‘ethnicity and compliant patient IAT’ adapted from the corresponding race IAT [31], and race and compliant patient IAT [26]. Adaptation included using NZ specific ethnic group categories (Māori and NZ European) with corresponding prototypical Māori and NZ European photographs [25]. The ‘ethnicity preference IAT’ required participants to sort prototypical photographs of Māori and NZ European people with word stimuli representing general concepts of ‘good’ and ‘bad’. The ‘ethnicity and compliant patient IAT’ required participants to sort the same prototypical photographs representing ethnicity with word stimuli representative of ‘compliant’ and ‘reluctant’ patients. The full set of word stimuli have been previously published [25].

IATs were built and hosted securely by Project Implicit® (www.projectimplicit.net) using a 7-block structure [32]. Participants linked directly to the IATs from the online study. Project Implicit provided raw latency data and calculated IAT D-scores (range − 2 to + 2) using standard guidelines [32]. Participants were excluded (*n* = 2) following standard criteria (more than 10% of responses classified as fast: < 300 ms response latency; and/or overall error rates greater than 30%) [32, 33]. A score of 0 indicated no implicit preference by ethnicity or no implicit compliance stereotype by patient ethnicity. Negative scores were pro-Māori (compared to NZ European) for that measure, and positive scores were pro-NZ European (compared to Māori).

#### Explicit bias measures

Ethnic preference for Māori compared to NZ European was examined on a 7-point scale (strongly/moderately/slightly prefer NZ European to Māori; like NZ European and Māori equally; slightly/moderately/strongly prefer Māori to NZ European) [9, 32]. Responses were reverse scored so that higher values indicated higher preference for NZ European. Warmth towards Māori and NZ European was assessed on separate 7-point scales from 1 = Feel LEAST WARM Toward This Group to 7 = Feel MOST WARM Toward This Group (adapted from [34]). Warmth difference scores were calculated for each participant (NZ European - Māori), with positive scores indicating greater reported warmth towards NZ European compared to Māori, and negative scores indicating greater warmth towards Māori.

#### Other covariates

We examined sociodemographic variables to check the vignette randomization process. These variables included age (in years from 20 to 30+), gender (male, female, other), ethnicity (prioritized as Māori, Pacific, Asian, Other, European) [35], nativity (born in NZ, born overseas), and self-reported socioeconomic position growing up (low, lower-middle, middle, upper-middle, high) [9]. Participants also completed the RAND 5-item Social Desirability (SD) response set [36] – scored from 0 to 5 (0 = low social desirability, 5 = high social desirability).

### Data analysis

All data were analyzed using R 3.1 (R Institute, Vienna, Austria). As some participants did not complete the entire study/testing procedure, analyses are restricted to those individuals participating in that stage of the study protocol. Responses were deleted for both the implicit and explicit bias measures if the respondent used a touch-based device (*n* = 10). The results section explicitly reports the number of participants contributing to each analysis.

Descriptive statistics are presented by randomized patient ethnicity vignette group to allow consideration for potential residual differences between groups following randomization. Sociodemographic variables are summarized with frequencies and percentages (for categorical variables) and medians and interquartile ranges (for continuous variables). Implicit and explicit bias measures are summarized with means and 95% confidence intervals (ethnicity preference IAT; ethnicity and compliant patient IAT; difference in warmth ratings assigned to NZ European and Māori, and ethnic preference).

Responses to vignette questions are summarized with means and 95% CI by randomized patient ethnicity for that vignette. For each vignette question, the difference in means by patient ethnicity is also presented (with 95% CI and *p*-value from an unpaired t-test).

We examined the association between implicit/explicit bias measures and each of the analyzed vignette questions using linear regression. As each participant only completed a vignette for one randomly assigned ethnicity, the difference in the slopes of these lines was compared using interaction terms in the linear regression models (interaction between assigned patient ethnicity and ethnic bias measure). These results are reported for each vignette measure as the estimate of slope in each of the two randomly assigned patient ethnicities (mean difference in vignette response for each one-unit difference in that bias measure, with 95% CI) alongside the difference in slopes between participants assigned the NZ European ethnicity vignette and participants assigned the Māori ethnicity vignette (with 95% CI, plus *p*-value for interaction term from the linear regression model).

To control for inflated Type I error rates from multiple hypothesis tests, we also considered results against a more stringent alpha value in analyses of clinical decision-making by patient ethnicity and its associations with measures of ethnic bias. We used a Bonferroni correction to allow an overall family-wise error rate within each vignette section of 0.05, which gave a more conservative alpha of 0.0125 for each comparison (0.05 / 4 = 0.0125), correcting for around 4 tests within each section. For example, for the mean differences between NZ European and Māori patients on vignette items, there were 3 vignette items for the CVD vignette and 4 for the depression vignette. For the regression analyses, there were analyses for 4 different bias measures within each vignette question.

## Results

Table 1 summarises participant characteristics overall and by randomized patient ethnicity (Māori or NZ European) for each vignette. Generally, characteristics of study participants were evenly distributed across vignette patient ethnicity groups. In the CVD vignette, Asian participants were more likely to receive a NZ European patient, and European participants were more likely to receive a Māori patient. There were also minor differences in distribution of Social Desirability (SD) scores by vignette patient ethnicity, although these were at the lower social desirability end of the scale.

Ethnic bias was apparent for implicit and explicit measures (Table 2). A mean ethnic preference IAT D-score of 0.39 (95% CI 0.33, 0.45) indicated an on average ‘moderate’ implicit preference for NZ Europeans compared to Māori. On average, participants also showed a ‘slight’ implicit association between the concept of a ‘compliant patient’ and NZ European patients compared with Māori patients (mean D-score 0.20, 95% CI 0.14, 0.26). On explicit measures we observed higher warmth toward NZ European compared to Māori (mean difference 0.19, 95% CI 0.06, 0.32) and a mean ethnic preference for NZ Europeans compared to Māori (mean 4.23, 95% CI 4.14, 4.32; a neutral score is 4). No systematic differences in ethnic bias were seen by randomly assigned patient ethnicity for each vignette.

**Table 2:** Participant ethnic bias results by vignette patient ethnicity

	CVD vignette ethnicity		Depression vignette ethnicity		
	NZ Euro patient	Māori patient	NZ Euro patient	Māori patient	Total
Participant bias Implicit/Explicit measure	n	n	n	n	n
	mean, 95% CI	mean, 95% CI	mean, 95% CI	mean, 95% CI	mean, 95% CI
IAT Ethnicity	95	103	105	93	198
preference^a^	0.43 (0.34, 0.51)	0.36 (0.28, 0.45)	0.37 (0.29, 0.46)	0.42 (0.33, 0.50)	0.39 (0.33, 0.45)
IAT Ethnicity &	75	69	71	73	144
compliant patient^a^	0.19 (0.11, 0.27)	0.21 (0.12, 0.30)	0.23 (0.14, 0.31)	0.17 (0.09, 0.25)	0.20 (0.14, 0.26)
Difference in warmth	118	115	122	111	233
ratings^b^ (NZE - Māori)	0.28 (0.09, 0.47)	0.10 (− 0.09, 0.28)	0.20 (0.04, 0.36)	0.18 (− 0.04, 0.40)	0.19 (0.06, 0.32)
Ethnic preference^c^	118	115	122	111	233
	4.26 (4.13, 4.40)	4.17 (4.04, 4.31)	4.23 (4.10, 4.36)	4.21 (4.06, 4.35)	4.22 (4.12, 4.31)

^a^D scores range from −2 to + 2. Negative scores show implicit preference or implicit higher compliance for Māori compared to European. Positive scores show implicit preference or implicit higher compliance for European compared to Māori

^b^7-point scale from 1 = LEAST WARM to 7 = MOST WARM for each ethnic group, mean difference is paired data

^c^7-point scale, response options reverse scored, 4 is neutral, above 4 preference for NZE, below 4 preference for Māori

There were no significant differences by patient ethnicity on diagnosis and management questions in the CVD vignette (Table 3). For the depression vignette, the likelihood of depression, severity of symptoms, and the estimated benefit of treatment did not significantly differ by patient ethnicity. For the recommended initial management approach item (response distributions in Additional file 1: Table S1), rankings were similar for the NZ European and Māori patient vignettes for the top ranked (encourage self-help strategies) and bottom ranked items (commence antidepressant and refer to specialist). For the NZ European patient vignette, the three middle options were ranked in order of preference from 2) recommend antidepressant; 3) refer to primary care brief intervention; and 4) refer to social support and counselling. These three responses received similar mean rankings for those who viewed the Māori patient vignette (i.e. they received equivalent management preference rankings on average). However, only referral to social support and counselling services was ranked significantly higher for the Māori compared to NZ European patient. Recommending an antidepressant was ranked higher for the NZ European than the Māori patient, although this was not significant under the more stringent alpha level of *p* < 0.0125.

**Table 3:** Responses to vignette diagnosis and management questions by patient ethnicity

Cardiovascular disease vignette	NZ European patient (*n* = 142)	Māori patient (*n* = 140)	Mean difference	*p*-value
Vignette questions	mean (95% CI)	mean (95% CI)	NZE - Māori (95% CI)	
Please assess the likelihood that Mr. [Wiremu’s/Williams’] pain is due to coronary artery disease^a^	3.59 (3.41, 3.76)	3.61 (3.44, 3.77)	− 0.02 (− 0.26, 0.22)	0.874
Using the information available, what would your recommendation be regarding thrombolysis for Mr. [Wiremu/Williams] when you discuss this case with your consultant?^b^	3.26 (3.08, 3.43)	3.25 (3.09, 3.41)	0.01 (− 0.23, 0.24)	0.966
If Mr. [Wiremu/Williams] refuses thrombolysis, how would you describe your subsequent management regarding thrombolysis?^c^	3.74 (3.61, 3.87)	3.59 (3.43, 3.76)	0.15 (− 0.07, 0.36)	0.176
Depression vignette	NZ European patient (*n* = 142)	Māori patient (*n* = 138)	Mean difference	*p*-value
Vignette questions	mean (95% CI)	mean (95% CI)	NZE - Māori (95% CI)	
Please assess the likelihood that Mr. [Tipene/Stephens’s] symptoms are due to depression^a^	3.89 (3.77, 4.00)	3.74 (3.61, 3.87)	0.15 (−0.03, 0.32)	0.096
Based on the information you have, how would you rate the severity of Mr. [Tipene/Stephens’s] symptoms?^d^	2.80 (2.72, 2.87)	2.88 (2.79, 2.96)	−0.08 (− 0.19, 0.03)	0.146
What initial management approach would you recommend? (options ranked from 1 to 5)^e^				
Encourage self-help strategies^f^	1.36 (1.20, 1.52)	1.36 (1.19, 1.52)	− 0.01 (− 0.23, 0.22)	0.955
Refer to social support and counselling services	3.28 (3.12, 3.44)	2.99 (2.83, 3.16)	− 0.29 (− 0.52, − 0.06)	0.012
Recommend anti-depressant treatment	2.73 (2.57, 2.89)	3.01 (2.84, 3.17)	0.28 (0.05, 0.50)	0.017
Refer to primary care brief intervention service^g^	3.06 (2.90, 3.22)	3.01 (2.84, 3.17)	−0.06 (− 0.28, 0.17)	0.626
Commence anti-depressant and refer to specialist mental health services	4.56 (4.40, 4.72)	4.64 (4.48, 4.80)	0.08 (−0.15, 0.31)	0.505
Please rate the likelihood that Mr. [Tipene/Stephens] will benefit from your selected initial management approach^h^	3.68 (3.56, 3.80)	3.64 (3.52, 3.77)	0.04 (− 0.14, 0.21)	0.684

^a^Response options (1 = very unlikely (< 20%), 2 = somewhat unlikely (20–40%), 3 = as likely as not (41–59%), 4 = somewhat likely (60–80%), 5 = very likely (> 80%))

^b^Response options reverse scored so (1 = would definitely NOT recommend thrombolysis, 2 = would probably NOT recommend thrombolysis, 3 = not sure, 4 = would probably recommend thrombolysis, 5 = would definitely recommend thrombolysis)

^c^Response options (1 = I would not try to persuade him any further to 5 = I would try very hard to persuade him)

^d^Response options (1 = none-slight, 2 = mild, 3 = moderate, 4 = severe)

^e^(mean rank, mean rank difference (NZE-Māori)

^f^(such as exercise, sleeping well, stress reduction, problem solving)

^g^(approximately 5 sessions of psychological therapy)

^h^Response options (1 = very unlikely, 2 = somewhat unlikely, 3 = as likely as not, 4 = somewhat likely, 5 = very likely)

Table 4 shows the associations between participants’ bias measures and vignette responses by patient ethnicity, alongside differences in associations by patient ethnicity. For CVD, no significant differences were found between slopes (and hence relationships between measures of ethnic bias and clinical decision-making) by patient ethnicity at the alpha of 0.0125. In the depression vignette, explicit preference for NZ Europeans was associated with students reporting an increased likelihood that NZ European patients would benefit from the selected initial management approach (slope 0.36, 95% CI 0.17, 0.54); while for Māori patients the slope was close to zero (slope 0.02, 95% CI -0.16, 0.20), and the differences in slopes by patient ethnicity was significant (difference in slope 0.34, 95% CI 0.08, 0.60; *p* = 0.011). No other vignette items showed a significantly different relationship with ethnic bias according to patient ethnicity.

**Table 4:** Associations between ethnic bias measures (implicit and explicit) and student diagnosis and treatment in vignettes by patient ethnicity

Cardiovascular disease vignette	Patient Described as:				Difference in	
Vignette question	NZ European		Māori		slope (NZE-Māori)	*p*-value
-bias measure	n	slope (95% CI)	n	slope (95% CI)	(95% CI)	
Please assess the likelihood that Mr. [Wiremu’s/Williams’] pain is due to coronary artery disease^a^						
-IAT ethnicity preference	95	−0.11 (− 0.63, 0.41)	103	− 0.15 (− 0.63, 0.32)	0.04 (− 0.66, 0.75)	0.906
-IAT ethnicity and compliant patient	75	0.17 (− 0.57, 0.91)	69	0.02 (− 0.67, 0.72)	0.15 (− 0.87, 1.16)	0.777
-explicit ethnic preference	118	0.03 (−0.23, 0.30)	115	− 0.08 (− 0.34, 0.18)	0.12 (− 0.26, 0.49)	0.539
-explicit ethnic warmth difference	118	0.01 (− 0.18, 0.19)	115	0.11 (−0.08, 0.30)	− 0.10 (− 0.37, 0.17)	0.456
Using the information available, what would your recommendation be regarding thrombolysis for Mr. [Wiremu/Williams] when you discuss this case with your consultant?^b^						
-IAT ethnicity preference	95	0.56 (0.10, 1.03)	103	−0.23 (− 0.65, 0.19)	0.79 (0.17, 1.42)	0.013
-IAT ethnicity and compliant patient	75	0.68 (0.00, 1.35)	69	−0.09 (− 0.72, 0.54)	0.77 (− 0.16, 1.69)	0.104
-explicit ethnic preference	118	−0.03 (− 0.28, 0.21)	115	− 0.28 (− 0.52, − 0.03)	0.24 (− 0.11, 0.59)	0.173
-explicit ethnic warmth difference	118	0.08 (− 0.09, 0.26)	115	−0.12 (− 0.30, 0.06)	0.20 (− 0.05, 0.45)	0.120
If Mr. [Wiremu/Williams] refuses thrombolysis, how would you describe your subsequent management regarding thrombolysis?^c^						
-IAT ethnicity preference	95	0.39 (−0.06, 0.84)	103	0.41 (0.00, 0.82)	−0.02 (− 0.63, 0.59)	0.946
-IAT ethnicity and compliant patient	75	0.47 (−0.15, 1.08)	69	0.27 (−0.31, 0.84)	0.20 (−0.64, 1.05)	0.634
-explicit ethnic preference	118	0.09 (−0.15, 0.32)	115	−0.04 (− 0.28, 0.19)	0.13 (− 0.20, 0.46)	0.438
-explicit ethnic warmth difference	118	0.07 (−0.10, 0.23)	115	−0.07 (− 0.24, 0.10)	0.13 (− 0.11, 0.37)	0.277
Depression vignette	Patient described as:				Difference in	
Vignette question	NZ European		Māori		slope (NZE-Māori)	*p*-value
-bias measure	n	slope (95% CI)	n	slope (95% CI)	(95% CI)	
Please assess the likelihood that Mr. [Tipene/Stephens’s] symptoms are due to depression^a^						
-IAT ethnicity preference	105	0.00 (− 0.33, 0.33)	93	−0.25 (− 0.60, 0.10)	0.25 (− 0.24, 0.73)	0.316
-IAT ethnicity and compliant patient	71	−0.09 (− 0.60, 0.42)	73	0.00 (− 0.53, 0.53)	−0.09 (− 0.83, 0.64)	0.801
-explicit ethnic preference	122	−0.02 (− 0.21, 0.17)	111	0.02 (− 0.17, 0.20)	−0.04 (− 0.30, 0.22)	0.768
-explicit ethnic warmth difference	122	0.06 (−0.09, 0.21)	111	0.03 (−0.09, 0.15)	0.03 (−0.16, 0.22)	0.754
Based on the information you have, how would you rate the severity of Mr. [Tipene/Stephens’s] symptoms?^d^						
-IAT ethnicity preference	105	0.14 (−0.08, 0.35)	93	−0.05 (− 0.28, 0.18)	0.18 (− 0.13, 0.50)	0.257
-IAT ethnicity and compliant patient	71	0.07 (−0.24, 0.38)	73	−0.21 (− 0.53, 0.11)	0.28 (− 0.16, 0.72)	0.212
-explicit ethnic preference	122	−0.01 (− 0.13, 0.11)	111	− 0.07 (− 0.19, 0.04)	0.06 (− 0.10, 0.23)	0.466
-explicit ethnic warmth difference	122	− 0.03 (− 0.12, 0.07)	111	0.05 (− 0.03, 0.12)	−0.07 (− 0.20, 0.05)	0.236
Please rate the likelihood that Mr. [Tipene/Stephens] will benefit from your selected initial management approach^e^						
-IAT ethnicity preference	105	0.04 (−0.31, 0.40)	93	−0.02 (− 0.40, 0.36)	0.06 (− 0.46, 0.59)	0.811
-IAT ethnicity and compliant patient	71	−0.17 (− 0.69, 0.35)	73	0.20 (− 0.34, 0.73)	−0.37 (− 1.11, 0.38)	0.330
-explicit ethnic preference	122	0.36 (0.17, 0.54)	111	0.02 (−0.16, 0.20)	0.34 (0.08, 0.60)	0.011
-explicit ethnic warmth difference	122	−0.19 (− 0.34, − 0.04)	111	−0.08 (− 0.20, 0.04)	−0.11 (− 0.30, 0.09)	0.285

^a^Response options (1 = very unlikely (< 20%), 2 = somewhat unlikely (20–40%), 3 = as likely as not (41–59%), 4 = somewhat likely (60–80%), 5 = very likely (> 80%))

^b^Response options reverse scored so (1 = would definitely NOT recommend thrombolysis, 2 = would probably NOT recommend thrombolysis, 3 = not sure, 4 = would probably recommend thrombolysis, 5 = would definitely recommend thrombolysis)

^c^Response options (1 = I would not try to persuade him any further to 5 = I would try very hard to persuade him)

^d^Response options (1 = none-slight, 2 = mild, 3 = moderate, 4 = severe)

^e^Response options (1 = very unlikely, 2 = somewhat unlikely, 3 = as likely as not, 4 = somewhat likely, 5 = very likely)

## Discussion

This is one of only two known studies to examine racial/ethnic bias and associations with cliical decision-making among medical students [12] and to our knowledge, is the first among medical students or any health professional group in New Zealand. The findings were mixed with regards to our hypotheses. Medical students demonstrated both implicit and explicit bias favouring NZ Europeans compared to Māori. However, we only found very limited evidence of differential decision-making by patient ethnicity, including differential associations between ethnic bias and clinical decision-making, with only two statistically significant findings among multiple tests (*n* = 35).

The mean pro-European implicit ethnic bias demonstrated in this study was similar to mean pro-white bias among US medical students using the Black-White race preference IAT [12, 16]. Implicit bias scores that associated compliance more closely with NZ European than Māori patients were similar to findings among US physicians [8, 10, 26]. The examination of ethnic bias in our study among a sample of final year medical students and for Māori compared to NZ European ethnic groups addresses identified research gaps in the international literature in terms of knowledge of health professional ethnic bias towards other minoritized ethnic groups and the use of a more nationally-representative sampling frame [7].

Encouragingly, we did not find evidence of ethnic bias being linked to differential clinical decision-making by patient ethnicity. The study of US physicians from which the CVD vignette here was adapted, found that increasing implicit preference for ‘Whites’ compared to ‘Blacks’ was associated with significantly increased likelihood of recommending thrombolysis for hypothetical ‘White’ patients, and a reduced tendency to recommend thrombolysis for hypothetical ‘Black’ patients [9]. While our study found similar directions in the relationship between implicit ethnic bias and clinical decision-making for NZ European compared to Māori patients, these were not significantly different. There is evidence of differential prescribing of antidepressants in New Zealand, with lower rates for Māori [37]. While this pattern was seen in the depression vignette, with lower ranking of the prescription of an anti-depressant for Māori compared to NZ European, this did not reach statistical significance. The only significant finding for the differential relationship between ethnic bias and patient management in the depression vignette was the association of explicit NZ European ethnic preference with increased perceived likelihood to benefit from the selected initial management approach for the NZ European patient but not for the Māori patient.

Both CVD and depression have known ethnic inequities in healthcare in NZ [37, 38]. However, despite the presence of ethnic bias, only one finding showed significant differential relationships between ethnic bias and clinical decision-making by patient ethnicity. This apparent lack of a relationship between racial/ethnic bias and clinical decision-making is seen in previous studies using similar methods [5, 39]. Amongst first year medical students in the US [12], pro-white implicit and explicit bias was demonstrated, but was not associated with differential responses to vignettes by patient race. A recent systematic review found that associations with race/ethnic bias have been more commonly reported in studies examining patient-provider interactions (e.g. patient perceptions of clinical encounters, physician communication styles) than in studies examining healthcare outcomes such as treatment decisions (as in our study), patient adherence and patient health outcomes [5]. For example, in a study of US primary care physicians, implicit race bias and implicit race and compliance stereotyping were associated with measures of poorer communication such as verbal dominance, and poorer patient perceptions of clinicians [8]. Implicit bias can be expressed in interpersonal interactions through subtle behaviours such as friendliness, body language, expressions and quality of speech that can impact on the quality of the encounter [29, 39]. These subtle expressions are less likely to be detected using vignette measures but may have subsequent impacts on patient care and inequities through pathways such as satisfaction and trust in healthcare, and adherence to recommended care [39, 40].

Other study limitations should also be considered. Participant responses to hypothetical vignette scenarios may not correspond to behaviour in actual clinical settings [41]. In particular, individuals’ implicit biases are more likely to be activated in situations with higher pressure and cognitive load that may be more common in real clinical situations [29, 39, 42]. It is possible that the use of words to describe ethnicity in clinical vignettes may elicit different responses than the use of visual images such as photographs. However, comparisons of implicit bias measures in New Zealand when using images or words to represent ethnicity have shown similar levels of bias in responses [43]. Although this study was designed to minimise the impact of social desirability, it is possible participants were aware of its purpose. Green et al. [9] found that participant awareness of the study purpose increased the likelihood of recommending thrombolysis to ‘Black’ patients. We are unable to examine the potential impact of this in our study although if it operates in a similar direction, our estimates are likely to be conservative. Ethnic bias was the focus of this study, and bias based on other attributes such as gender were standardised but not examined in the vignettes. Finally, the response rate and the absolute study numbers were lower than expected, which may impact on generalisability and study power. The distribution of participants by age, gender and ethnicity was similar to final year medical student demographic profiles, which is reassuring for the generalisability of initial questions such as vignette responses by patient ethnicity. Dropout of participants as they progressed through the online study may bias analyses using data from later questions although in analyses of vignette responses by patient ethnicity in a restricted sample of those who completed the questionnaire, patterning of results were similar.

## Conclusions

To our knowledge, this is the first study of its kind among any health professional group in New Zealand and one of only a few among medical students internationally. The findings extend the evidence on health professional racial/ethnic bias beyond the current dominance of US-based studies, providing information on bias against other ethnic groups, including another indigenous population. While on average final year medical students expressed ethnic bias favoring NZ European patients compared to Māori, evidence of links to clinical decision-making were not found. Further research is required into the ways ethnic bias may be expressed in healthcare encounters, and ethnic bias among other groups including different student year groups, faculty, and physicians.

Our findings demonstrate the need to address ethnic bias in medical student education in New Zealand, particularly in relation to indigenous health. This has implications for medical education and broader healthcare environments. Ethnic bias training that aims to understand, identify, mitigate and reduce ethnic bias should be included in formal medical school curricula. This is supported by evidence that inclusion of ethnic bias in formal curricula by trained instructors and improving student confidence in providing care for minority patients are associated with reduced implicit racial/ethnic bias [44]. Additional efforts are also needed to address aspects of the hidden curriculum and clinical environments that may increase ethnic bias [44] and to introduce health system factors that may mitigate expression of ethnic bias. Finally, the broader context of societal racism within which individual ethnic bias develops requires addressing [3, 39].

## Additional file

Additional file 1: Table S1. Distribution of responses to vignette diagnosis and management questions by patient ethnicity. Distribution of responses to vignette diagnosis and management questions by patient ethnicity. (DOCX 29 kb)

## Abbreviations

BDMM: Bias and Decision-Making in Medicine
CI: Confidence interval
CVD: Cardiovascular disease
E4E: Educating for Equity
IAT: Implicit association test
MI: Myocardial Infarction
NZ: New Zealand
NZE: New Zealand European
SD: Social Desirability
US: United States
