# Supplementary material for: Ethnic bias and clinical decision-making among New Zealand medical students: an observational study
Source: BMC Med Educ. 2018 Jan 23;18:18. doi: 10.1186/s12909-018-1120-7 (PMC5782368; doi:10.1186/s12909-018-1120-7)
Supplement: Additional file 1: Table S1. — Distribution of responses to vignette diagnosis and management questions by patient ethnicity. Distribution of responses to vignette diagnosis and management questions by patient ethnicity. (DOCX 29 kb) [file 12909_2018_1120_MOESM1_ESM.docx]

**Table S1: Distribution of responses to vignette diagnosis and management questions by patient ethnicity**

|  | Patient Described as | |
| --- | --- | --- |
| **Cardiovascular disease vignette items and response** | NZ European (n=145) | Māori (n=142) |
| **options** | n (%) | n (%) |
|  |  |  |
| Please assess the likelihood that Mr [Wiremu/Williams's] pain is due to coronary artery disease | |  |
| 1 = very unlikely (<20%) | 2 (1) | 1 (1) |
| 2 = somewhat unlikely (20-40%) | 29 (20) | 27 (19) |
| 3 = as likely as not (41-59%) | 25 (17) | 24 (17) |
| 4 = somewhat likely (60-80%) | 60 (41) | 65 (46) |
| 5 = very likely (>80%) | 29 (20) | 25 (18) |
| *Not reported* | 0 (NA) | 0 (NA) |
|  |  |  |
| Using the information available, what would your recommendation be regarding thrombolysis for Mr [Wiremu/Williams] when you discuss this case with your consultant?^a^ | | |
| 1 = would definitely NOT recommend thrombolysis | 6 (4) | 5 (4) |
| 2 = would probably NOT recommend thrombolysis | 34 (23) | 30 (21) |
| 3 = not sure | 38 (26) | 37 (26) |
| 4 = would probably recommend thrombolysis | 51 (35) | 61 (44) |
| 5 = would definitely recommend thrombolysis | 16 (11) | 7 (5) |
| *Not reported* | 0 (NA) | 2 (NA) |
|  |  |  |
| If Mr [Wiremu/Williams] refuses thrombolysis, how would you describe your subsequent management regarding thrombolysis? | | |
| 1 = I would try not to persuade him any further | 1 (1) | 6 (4) |
| 2 | 9 (6) | 14 (10) |
| 3 | 36 (25) | 31 (22) |
| 4 | 76 (54) | 69 (49) |
| 5 = I would try very hard to persuade him | 20 (14) | 20 (14) |
| *Not reported* | 3 (NA) | 2 (NA) |
|  |  |  |
|  | Patient Described as: | |
| **Mental health vignette questions and response options** | NZ European (n=142) | Māori (n=138) |
|  | n (%) | n (%) |
|  |  |  |
| Please assess the likelihood that Mr [Tipene’s/Stephens'] symptoms are due to depression | |  |
| 1 = very unlikely (<20%) | 1 (1) | 2 (1) |
| 2 = somewhat unlikely (20-40%) | 5 (4) | 10 (7) |
| 3 = as likely as not (41-59%) | 22 (15) | 23 (17) |
| 4 = somewhat likely (60-80%) | 95 (67) | 90 (65) |
| 5 = very likely (>80%) | 19 (13) | 13 (9) |
| *Not reported* | 0 (NA) | 0 (NA) |
|  |  |  |
| Based on the information you have, how would you rate the severity of Mr [Tipene/Stephens's] symptoms? | |  |
| 1 = none-slight | 0 (0) | 0 (0) |
| 2 = mild | 31 (22) | 26 (19) |
| 3 = moderate | 109 (77) | 103 (75) |
| 4 = severe | 2 (1) | 9 (7) |
| *Not reported* | 0 (NA) | 0 (NA) |
|  |  |  |
| Please rate the likelihood that Mr [Tipene/Stephens] will benefit from your selected initial management approach | |  |
| 1 = very unlikely | 0 (0) | 0 (0) |
| 2 = somewhat unlikely | 11 (8) | 11 (8) |
| 3 = as likely as not | 35 (25) | 37 (27) |
| 4 = somewhat likely | 83 (59) | 80 (58) |
| 5 = very likely | 12 (9) | 10 (7) |
| *Not reported* | 1 (NA) | 0 (NA) |

^a^responses have been reverse scored
